# Pitfalls and tips for lumenless lead extraction inserted deep within the ventricular septum

**DOI:** 10.1002/ccr3.8718

**Published:** 2024-04-24

**Authors:** Nobunari Tomura, Hirokazu Shiraishi, Masahiro Makino, Jun Munakata, Satoshi Shimoo, Hibiki Iwakoshi, Tetsuro Nishimura, Takashi Ohkura, Keitaro Senoo, Satoaki Matoba

**Affiliations:** ^1^ Department of Cardiovascular Medicine, Graduate School of Medical Sciences Kyoto Prefectural University of Medicine Kyoto Japan

**Keywords:** lead extraction, left bundle branch area pacing, lumenless pacemaker lead, rotation dilator sheath, ventricular septum

## Abstract

**Key Clinical Message:**

This case highlights the pitfalls and provides tips for the extraction of deeply implanted lumenless leads, and encourages careful lead selection in the current era of widespread left bundle branch area pacing.

**Abstract:**

The extraction of cardiovascular implantable electronic device leads is sometimes complicated. We describe a case with difficult but successful extraction of SelectSecure, a lumenless permanent pacemaker lead, implanted deep in the ventricular septum, highlighting its pitfalls and tips in the current era of left bundle branch area pacing.

## Graphical abstract

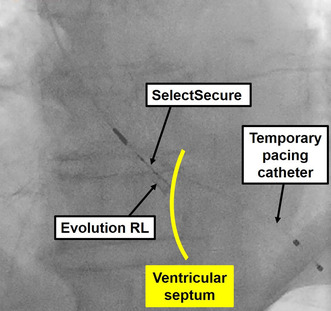

The pitfalls and provides tips for the extraction of deeply implanted lumenless leads in the ventricular septum with using Evolution mechanical sheath

## 1 INTRODUCTION

Removing cardiovascular implantable electronic device (CIED) leads while treating individuals with CIED infection or lead failure is crucial. However, the lead type, dwell time, and position can complicate this procedure, leading to significant complications.^1^ We report a case of difficult but successful extraction of a lumenless permanent pacemaker lead implanted deep in the ventricular septum.

## 2 CASE HISTORY/EXAMINATION

An 84‐year‐old man presented to the emergency department of an affiliated hospital with syncope. The patient underwent implantation of a dual‐chamber transvenous pacemaker 22 months prior. Right ventricular (RV) septal pacing was performed with SelectSecure (Medtronic Inc., Minneapolis, MN, USA) to treat heart failure complicated with bradycardia and atrial fibrillation (AF). The patient underwent catheter ablation twice after pacemaker implantation, but AF remained. Pacemaker interrogation detected normal impedance, but there was an increase in the threshold of the RV lead, resulting in loss of ventricular capture. Because pacing failure continued despite adjustment of the pacemaker settings, the patient was referred to our hospital for extraction of the existing system and replacement with a leadless pacemaker.

**FIGURE 1:**
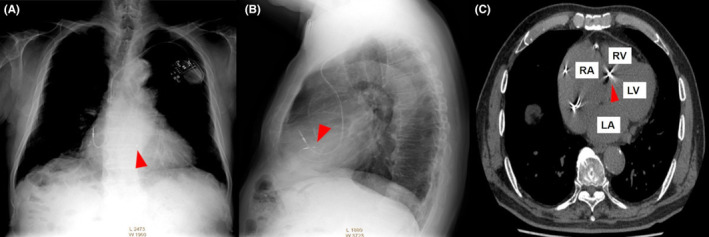
Chest X‐ray (A, B) and CT (C) showing the RV lead implanted into the basal RV septum (arrowheads). CT, computed tomography; RV, right ventricular.

**FIGURE 2:**
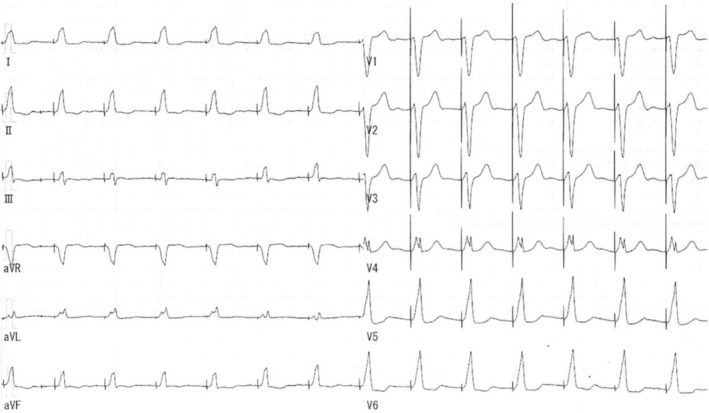
Twelve‐lead electrocardiogram showing AF and septal pacing with a QRS duration of 151 ms. AF, atrial fibrillation.

On admission, the patient's physical examination revealed no abnormalities. On chest X‐ray and computed tomography, an implanted RV lead was seen in the basal RV septum (Figure 1). Twelve‐lead electrocardiogram demonstrated AF and ventricular pacing with a QRS duration of 151 ms (Figure 2). Echocardiogram showed preserved left ventricular ejection fraction without dyssynchrony.

## 3 METHODS

**FIGURE 3:**
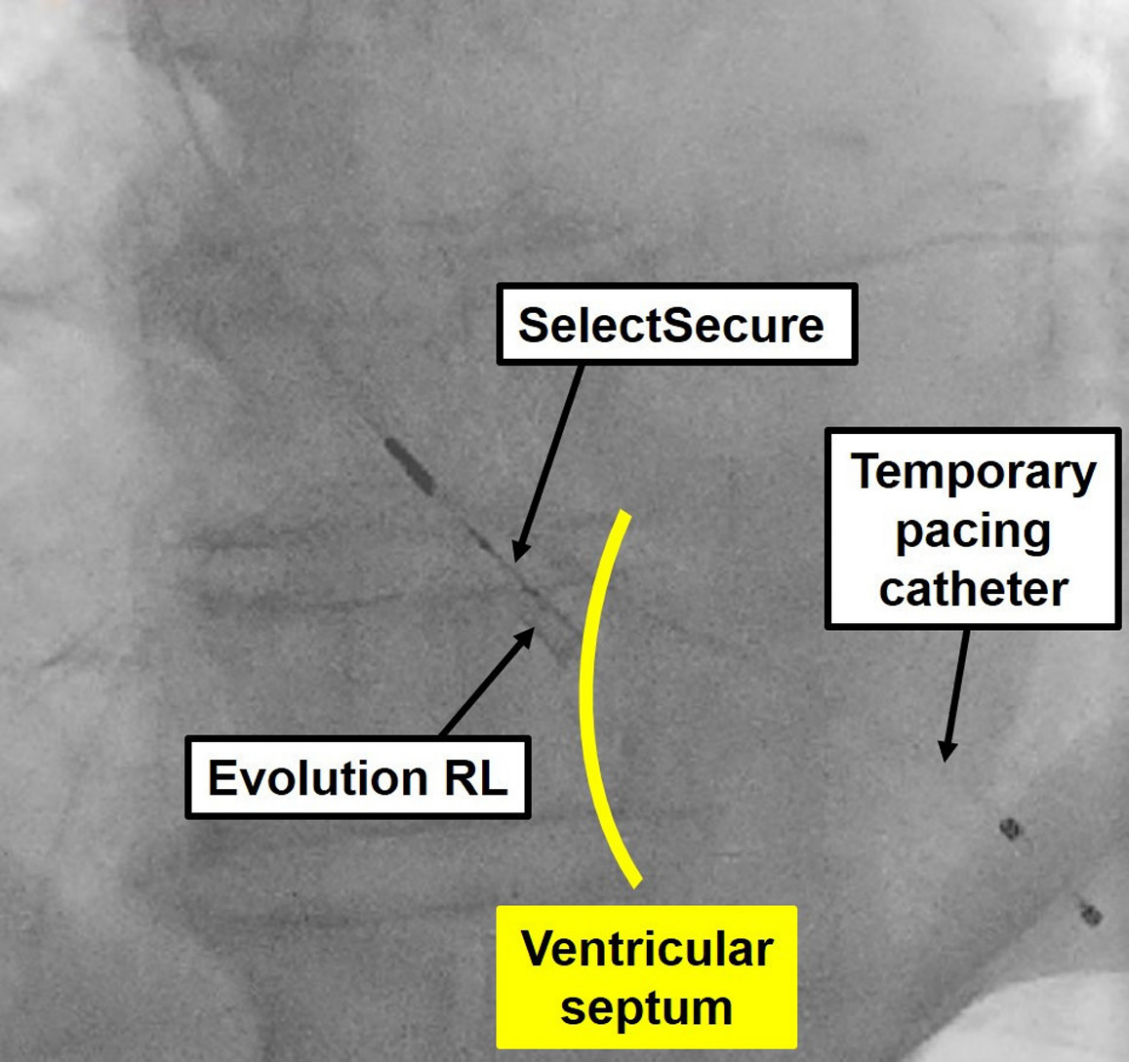
Angiogram showing traction of the RV lead with an Evolution sheath pressed against the ventricular septum. RV, right ventricular.

The procedure for lead extraction in the hybrid operating room was as follows. After gaining access to the femoral vein and artery, a temporary RV pacing catheter was inserted. After pacemaker pocket incision, the pacemaker leads were disconnected from the generator. The right atrial lead (CapSureFIX Novus; Medtronic Inc.) was easily removed by manual traction after screw‐out using a standard stylet. However, the RV lead could not be removed by counterclockwise turning of the lead body and traction. After cutting the body of the lead using sturdy scissors, we connected a Bulldog lead extender (Cook Medical, Bloomington, IN, USA) to its inner conductor cable. The lead extender was then fixed in place with silk sutures. Next, we dissected the adhesions from the subclavian vein to the superior vena cava using a 9‐F Evolution RL Rotation dilator sheath (Cook Medical) under fluoroscopic guidance. Finally, we extracted the RV lead completely by traction with the sheath pressed against the ventricular septum (Figure 3; Video S1).

## 4 CONCLUSION AND RESULTS

A leadless pacemaker was implanted because a preoperative contrast scan confirmed right subclavian vein occlusion. Postoperative echocardiogram showed neither pericardial effusion nor ventricular septal perforation. The patient was discharged 7 days postoperatively without any sequelae.

## 5 DISCUSSION

Owing to its small diameter (4.1 F) and use of a steerable or preformed sheath for deployment, the SelectSecure lead is useful for transvenous pacing in patients with pediatric and adult congenital heart disease.^2^ The lead has an exposed, non‐retractable helix and no lumen, increasing its composite pull strength. Although some studies have demonstrated the ease and safety of its extraction,^3^, ^4^ these design features require specific considerations in terms of the extraction strategy (Table S1).^5^, ^6^, ^7^

In addition, the use of the SelectSecure lead for left bundle branch area (LBBA) pacing as a substitute for His bundle pacing to deliver cardiac resynchronization treatment or prevent RV pacing has recently attracted much attention. His bundle pacing has limitations, such as a high pacing threshold and a high lead dislocation rate. These limitations are addressed by LBBA pacing, which advances the lead into the ventricular septum to reach the LBBA.^8^

In our case, the SelectSecure was inserted deep within the ventricular septum, but 12‐lead electrocardiogram did not show an LBBA pacing pattern. We had no choice but to use the inner cable of the lead like a locking stylet using a lead extender because the locking stylet could not be inserted. Of note, even though the fibrosis of the SelectSecure helix was not expected to be advanced because of the relatively short period from implantation to extraction (22 months), it was difficult to release its adhesion to the myocardium of the ventricular septum. Based on the operative findings and normal lead impedance on admission, the cause of the increased RV lead threshold was thought to be the development of fibrosis surrounding the electrode tip. Although the Evolution sheath was pressed softly against the ventricular septum and the reaction mechanism was used to successfully remove the lead, a catastrophe could have resulted if an inappropriate procedure had been used. We consider that this strategy of removing the SelectSecure lead implanted deep into the ventricular septum is useful for lead extraction in LBBA pacing.

With the popularity of LBBA pacing, it is anticipated that an increasing number of patients with implanted SelectSecure leads may eventually need to undergo lead removal. The present case highlights the pitfalls and provides tips for the extraction of deeply implanted lumenless leads, and encourages careful lead selection with the goal of removal.

## AUTHOR CONTRIBUTIONS

**Nobunari Tomura:** Conceptualization; writing – original draft; writing – review and editing. **Hirokazu Shiraishi:** Conceptualization; supervision; writing – original draft; writing – review and editing. **Masahiro Makino:** Investigation; validation; writing – review and editing. **Jun Munakata:** Investigation; validation; writing – review and editing. **Satoshi Shimoo:** Investigation; validation; writing – review and editing. **Hibiki Iwakoshi:** Investigation; validation; writing – review and editing. **Tetsuro Nishimura:** Investigation; validation; writing – review and editing. **Takashi Ohkura:** Investigation; validation; writing – review and editing. **Keitaro Senoo:** Investigation; validation; writing – review and editing. **Satoaki Matoba:** Conceptualization; supervision; writing – original draft; writing – review and editing.

## FUNDING INFORMATION

No funding was secured for this study.

## CONFLICT OF INTEREST STATEMENT

The authors declare that they have no conflicts of interest.

## ETHICS APPROVAL STATEMENT

This paper was approved by the Kyoto Prefectural University of Medicine Ethics Committee.

## CONSENT

Written informed consent was obtained from the patient to publish this report in accordance with the journal's patient consent policy.

## Supporting information


Table S1

Video S1

Data S1

## Data Availability

All relevant data supporting the conclusions of this article are included within the article.
