# Supplementary material for: Pitfalls and tips for lumenless lead extraction inserted deep within the ventricular septum
Source: Clin Case Rep. 2024 Apr 24;12(5):e8718. doi: 10.1002/ccr3.8718 (PMC11043091; doi:10.1002/ccr3.8718)
Supplement: Supplementary file 1 — Table S1 [file CCR3-12-e8718-s003.docx]

**Table S1. Summary of reports regarding Select Secure leads extraction**

|  | Shepherd E, et al.  (n=22) | Garnreiter J, et al.  (n=9) | Migliore F, et al.  (n=1) | Krainski F, et al.  (n=1) | Boczar K, et al.  (n=1) |
| --- | --- | --- | --- | --- | --- |
| Age, years | 17.6±10.5 | Unknown | 83 | 14 | 66 |
| Male, n | 11 | Unknown | 1 | 1 | 1 |
| Congenital heart disease, n | 12 | Unknown | 0 | 1 | 0 |
| Lead dwell time, years | 4.1±2.6 | 1.4±1.5 | 14 | 5 | 1 |
| Lead position | Unknown | Unknown | His bundle | RV apex | His bundle |
| Indication | | | | | |
| Infection, n | 1 | 1 | 1 | - | - |
| Lead failure, n | 3 | 4 | - | 1 | - |
| Other, n | 18 | 4 | - | - | 1 |
| Extraction method | | | | | |
| Manual traction, n | 9 | 9 | - | - | - |
| Mechanical sheath, n | 7 | 0 | - | - | 1 |
| Evolution sheath, n | 6 | 0 | 1 | - | - |
| Laser sheath, n | 0 | 0 | - | 1 | - |
| Complex extraction* | 1 | 0 | 0 | 0 | 1 |
| Major Complications** | 0 | 0 | 0 | 0 | 0 |

* Composite of femoral extraction and partial extraction

**Composite of pericardial effusion, surgical extraction and death

RV: right ventricle
