# Supplementary material for: Pitfalls and tips for lumenless lead extraction inserted deep within the ventricular septum
Source: Clin Case Rep. 2024 Apr 24;12(5):e8718. doi: 10.1002/ccr3.8718 (PMC11043091; doi:10.1002/ccr3.8718)
Supplement: Supplementary file 3 — Data S1 [file CCR3-12-e8718-s001.docx]

**Supplemental caption**

Video S1 Angiogram showing RV lead extraction
RV = right ventricular
